# (2-Amino­ethan-1-aminium-κ*N*^2^)tri­chlorido­zinc(II)

**DOI:** 10.1107/S2414314625007710

**Published:** 2025-09-05

**Authors:** Aboubacar Diop, Daouda Ndoye, Paul Tinnemans, Ennio Zangrando, Cheikh Abdoul Khadir Diop

**Affiliations:** a Inorganic and Analytical Chemistry Laboratory, Department of Chemistry, Faculty of Science and Technology, Cheikh Anta Diop University, Dakar, Senegal; b Institute for Molecules & Materials (IMM), Solid State Chemistry, Faculty of Science, Radboud University, Nijmegen, The Netherlands; c Departement of Chemical and Pharmaceutical Sciences, University of Trieste, Trieste, Italy; d Inorganic and Analytical Chemistry Laboratory, Departement of Chemistry, Faculty of Science and Technology, Cheikh Anta Diop University, Dakar, Senegal; Vienna University of Technology, Austria

**Keywords:** crystal structure, zinc(II), zwitterionic complex, ethyl­enedi­amine

## Abstract

The mononuclear zwitterionic Zn^II^ complex, [ZnCl_3_(C_2_H_9_N_2_)], contains a monodentate 2-ammonio­ethyl­amine moiety and three chloride anions coordinating to the central zinc atom in form of a distorted tetra­hedron. The molecular packing shows amine and ammonium groups inter­acting through hydrogen-bonding inter­actions with chlorine atoms of symmetry-related complexes to form a supra­molecular framework structure.

## Abstract 2

The coordination environment of the central Zn^II^ atom in the zwitterionic mol­ecular title complex is distorted tetra­hedral, consisting of one N and three Cl atoms.

## Graphical abstract

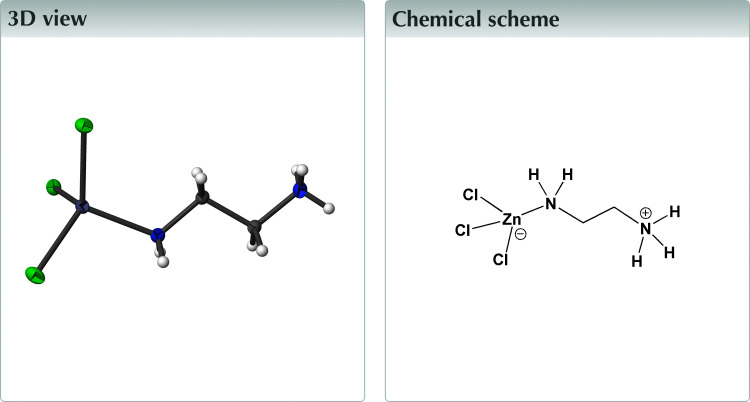

## Structure description

Zwitterionic tri­chlorido­zinc(II) complexes with the metal additionally ligated in a monodentate manner by a protonated organic mol­ecule are known (Clemente *et al.*, 2002 ▸; Maixner & Zachová, 1993 ▸; Purnell & Hodgson, 1976 ▸; Sheldrick, 1982 ▸; Steffen & Palenik, 1978 ▸; Zhu *et al.*, 2002 ▸), including a protonated quinine ligand (Kang *et al.*, 2013 ▸). The commonly used ethyl­enedi­amine (en) ligand acts primarily as a chelating ligand, and monodentate (Fanshawe *et al.*, 2000 ▸) or bridging forms (Çolak *et al.*, 2008 ▸) of en are rather rare. An example of a protonated and monodentate en ligand has been reported within a germanotungstate polyanion composed of two [GeW_9_O_34_]^10–^ moieties sandwiching a rhomboid-like Zn_4_ cluster, in which two central Zn^II^ atoms are coordinated by the N atom of the non-protonated amino group (Wang *et al.* 2010 ▸). In this context, we synthesized the title complex [ZnCl_3_(C_2_H_9_N_2_)] using en, hydro­chloric acid and zinc chloride as starting materials.

**Figure 1:**
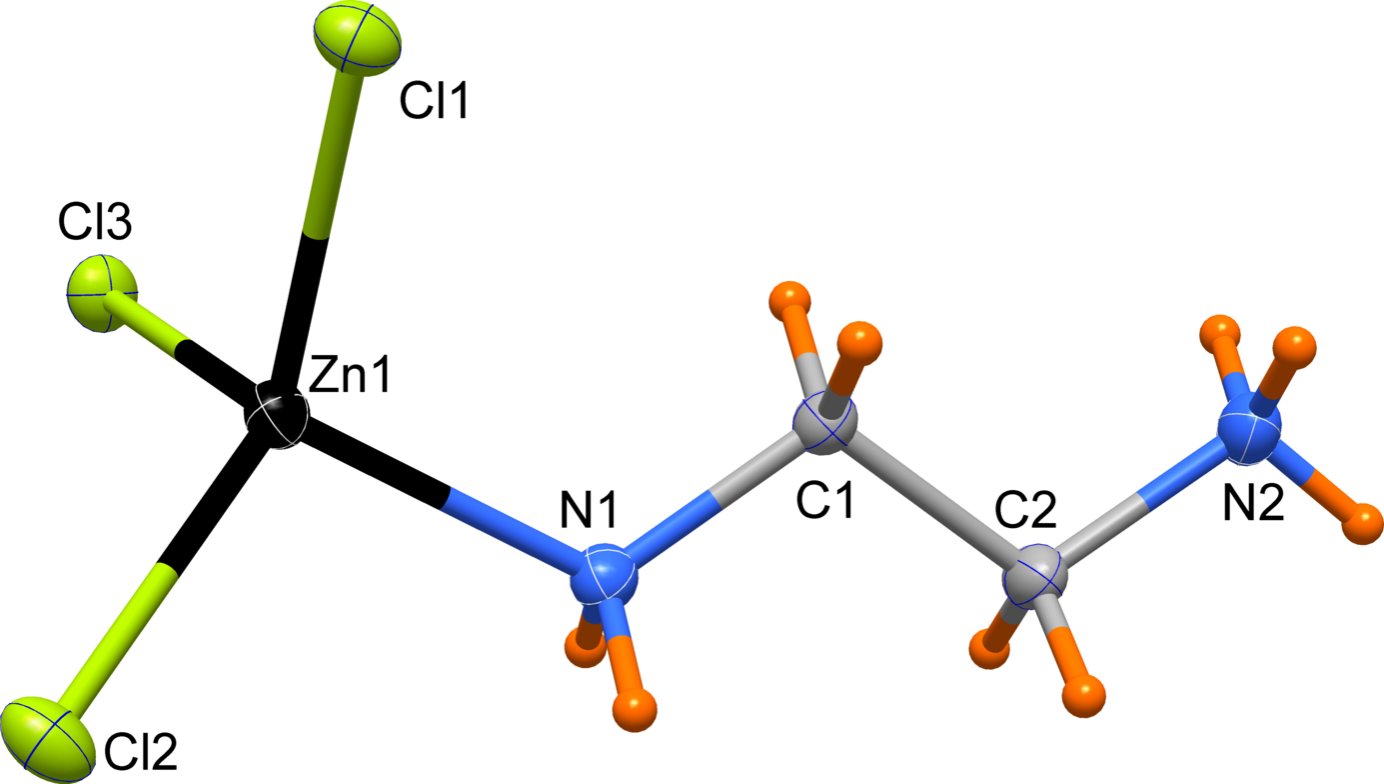
Mol­ecular structure of the title complex with displacement ellipsoids drawn at the 50% probability level.

The mol­ecular structure of [ZnCl_3_(C_2_H_9_N_2_)] is characterized by a protonated en ligand, monodentately binding through the amine N atom (N1) to the central Zn^II^ atom. The tetra­hedral coordination environment is completed by three Cl^−^ ligands (Fig. 1 ▸). The Zn—N bond length is 2.0345 (10) Å, much shorter than the values found in the Zn^II^ complexes with chelating ethyl­endi­amine (en) ligands, for example [Zn(en)(acetate)_2_] (Kim *et al.*, 2007 ▸) where the Zn—N distance is 2.0784 (16) Å, or in the complex octa­hedral cation [Zn(en)_3_]^2+^ (Cheng *et al.*, 2008 ▸) with distances between 2.159 (2) and 2.220 (2). The present Zn—N bond length is also considerably shorter than in the germanotungstate cluster comprising a Zn^II^ atom bound to a monodentate en ligand [2.121 (16) Å; Wang *et al.*, 2010 ▸]. The three Zn—Cl bond lengths in the title complex range from 2.2600 (3) to 2.2686 (3) Å, which is comparable to those previously reported in the complexes having a ZnCl_3_ moiety mentioned above. The present Cl—Zn—Cl and N—Zn—Cl bond angles vary from 104.52 (3) to 115.969 (12)°, indicating a considerable distortion from an ideal tetra­hedron. The protonated en ligand has an *anti-*conformation with an N1—C1—C2—N2 torsion angle of 178.18 (10)°.

**Figure 2:**
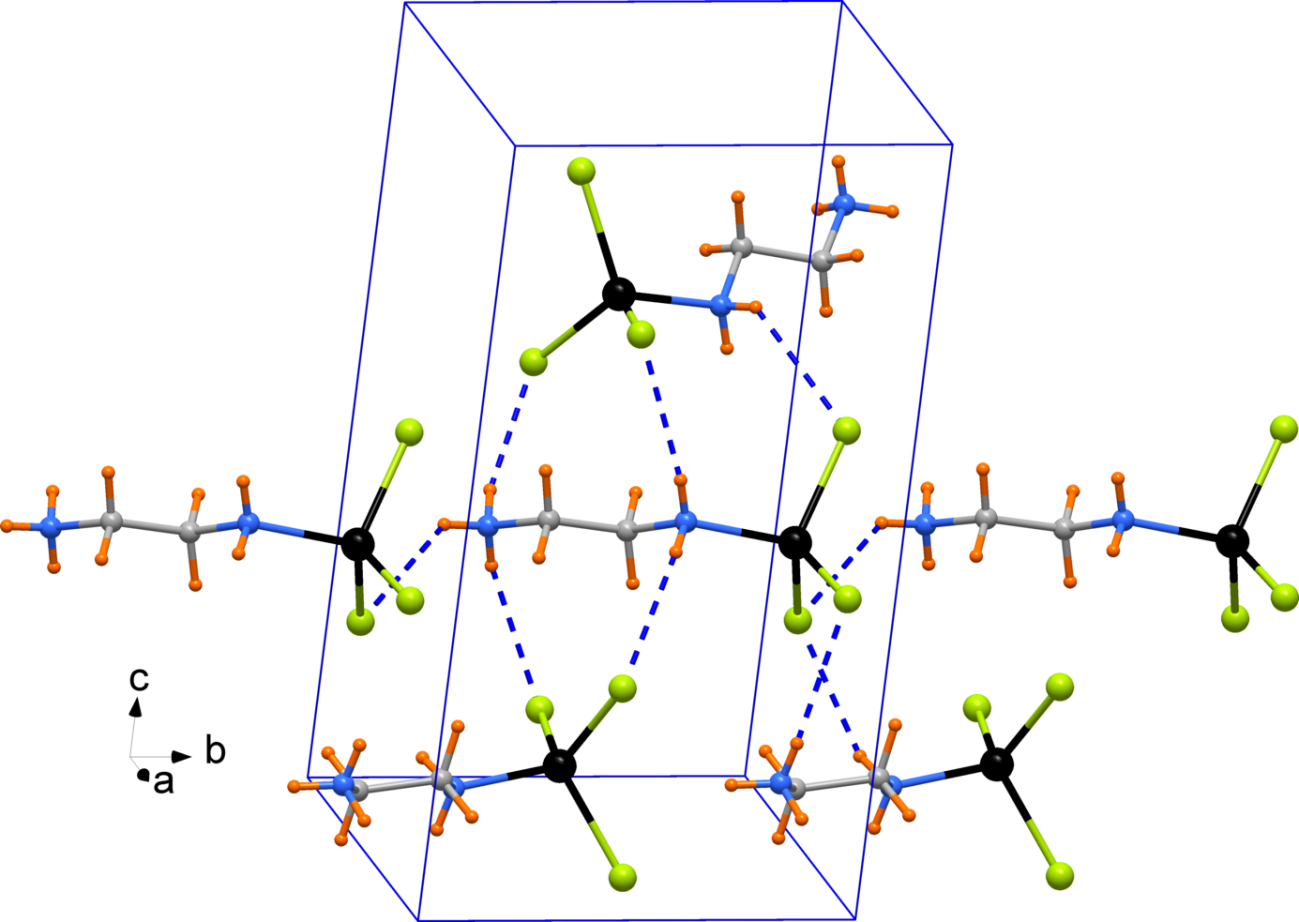
Partial view of the molecular packing in the crystal of the title complex with N—H⋯Cl hydrogen bonds indicated as dashed lines

The molecular packing is stabilized by an intricate framework of inter­molecular N—H⋯Cl hydrogen bonds involving both the amine (N1) and ammonium (N2) groups as donors and all three Cl ligands as acceptor atoms (Table 1 ▸). Part of the crystal packing is illustrated in Fig. 2 ▸.

## Synthesis and crystallization

The title complex was obtained by addition of a methano­lic solution (10 ml) of ZnCl_2_ (0.136 g, 1 mmol) to a flask containing 10 ml of a methano­lic solution of ethyl­enedi­amine, C_2_H_8_N_2_ (0.06 g, 1 mmol) and 5 ml of hydro­chloric acid HCl (1 *N*). The resulting mixture was stirred for 2 h at room temperature. A clear solution was obtained and left to evaporate slowly at room temperature, leading to colorless single crystals suitable for single-crystal X-ray diffraction after 24 h.

## Refinement

Crystal data, data collection and structure refinement details are summarized in Table 2 ▸.

## Supplementary Material

Crystal structure: contains datablock(s) I. DOI: 10.1107/S2414314625007710/wm4235sup1.cif

Structure factors: contains datablock(s) I. DOI: 10.1107/S2414314625007710/wm4235Isup3.hkl

CCDC reference: 2483701

Additional supporting information:  crystallographic information; 3D view; checkCIF report

## Figures and Tables

**Table 1:** Hydrogen-bond geometry (Å, °)

*D*—H⋯*A*	*D*—H	H⋯*A*	*D*⋯*A*	*D*—H⋯*A*
N1—H1*A*⋯Cl1^i^	0.883 (18)	2.636 (17)	3.3733 (11)	141.7 (14)
N1—H1*B*⋯Cl2^ii^	0.828 (17)	2.678 (18)	3.4620 (11)	158.6 (17)
N2—H2*A*⋯Cl3^ii^	0.85 (2)	2.46 (2)	3.2188 (12)	149.2 (17)
N2—H2*B*⋯Cl1^iii^	0.87 (2)	2.566 (19)	3.2449 (11)	135.2 (16)
N2—H2*C*⋯Cl2^i^	0.81 (2)	2.70 (2)	3.4526 (12)	154.2 (19)

Symmetry codes: (i) 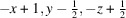; (ii) 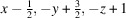; (iii) 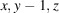.

**Table 2:** Experimental details

Crystal data	
Chemical formula	[ZnCl_3_(C_2_H_9_N_2_)]
*M*_r_	232.83
Crystal system, space group	Orthorhombic, *P*2_1_2_1_2_1_
Temperature (K)	150
*a*, *b*, *c* (Å)	6.6669 (4), 8.1192 (4), 14.7559 (7)
*V* (Å^3^)	798.74 (7)
*Z*	4
Radiation type	Mo *K*α
μ (mm^−1^)	3.99
Crystal size (mm)	0.24 × 0.13 × 0.11

Data collection	
Diffractometer	Bruker *APEX* CCD area-detector
Absorption correction	Multi-scan (*SADABS*; Krause *et al.*, 2015 ▸)
*T*_min_, *T*_max_	0.407, 0.520
No. of measured, independent and observed [*I* > 2σ(*I*)] reflections	11232, 3036, 3017
*R*_int_	0.020
(sin θ/λ)_max_ (Å^−1^)	0.770

Refinement	
*R*[*F*^2^ > 2σ(*F*^2^)], *wR*(*F*^2^), *S*	0.012, 0.026, 1.12
No. of reflections	3036
No. of parameters	101
H-atom treatment	Only H-atom coordinates refined
Δρ_max_, Δρ_min_ (e Å^−3^)	0.29, −0.33
Absolute structure	Refined as an inversion twin Parsons *et al.*, 2013 ▸]
Absolute structure parameter	0.374 (5)

Computer programs: *SMART* and *SAINT* (Bruker, 2000 ▸), *SHELXT* (Sheldrick, 2015*a* ▸), *SHELXL* (Sheldrick, 2015*b* ▸), *DIAMOND* (Brandenburg & Putz, 1999 ▸) and *WinGX* (Farrugia, 2012 ▸).

## full crystallographic data

### (2-Aminoethan-1-aminium-κN2)trichloridozinc(II) Crystal data

**Table:**

[ZnCl_3_(C_2_H_9_N_2_)]	*D*_x_ = 1.936 Mg m^−^^3^
*M**_r_* = 232.83	Mo *K*α radiation, λ = 0.71073 Å
Orthorhombic, *P*2_1_2_1_2_1_	Cell parameters from 7854 reflections
*a* = 6.6669 (4) Å	θ = 1.9–25.0°
*b* = 8.1192 (4) Å	µ = 3.99 mm^−^^1^
*c* = 14.7559 (7) Å	*T* = 150 K
*V* = 798.74 (7) Å^3^	Block, colorless
*Z* = 4	0.24 × 0.13 × 0.11 mm
*F*(000) = 464	

### (2-Aminoethan-1-aminium-κN2)trichloridozinc(II) Data collection

**Table:**

Bruker APEX CCD area-detector diffractometer	3036 independent reflections
Radiation source: fine-focus sealed tube	3017 reflections with *I* > 2σ(*I*)
Graphite monochromator	*R*_int_ = 0.020
ω scans	θ_max_ = 33.2°, θ_min_ = 2.8°
Absorption correction: multi-scan (*SADABS*; Krause *et al.*, 2015)	*h* = −9→10
*T*_min_ = 0.407, *T*_max_ = 0.520	*k* = −12→12
11232 measured reflections	*l* = −22→22

### (2-Aminoethan-1-aminium-κN2)trichloridozinc(II) Refinement

**Table:**

Refinement on *F*^2^	Hydrogen site location: difference Fourier map
Least-squares matrix: full	Only H-atom coordinates refined
*R*[*F*^2^ > 2σ(*F*^2^)] = 0.012	*w* = 1/[σ^2^(*F*_o_^2^) + (0.0075*P*)^2^ + 0.0649*P*] where *P* = (*F*_o_^2^ + 2*F*_c_^2^)/3
*wR*(*F*^2^) = 0.026	(Δ/σ)_max_ = 0.002
*S* = 1.12	Δρ_max_ = 0.28 e Å^−^^3^
3036 reflections	Δρ_min_ = −0.33 e Å^−^^3^
101 parameters	Absolute structure: Refined as an inversion twin Parsons *et al.*, 2013]
0 restraints	Absolute structure parameter: 0.374 (5)

### (2-Aminoethan-1-aminium-κN2)trichloridozinc(II) Special details

**Table:**

Geometry. All esds (except the esd in the dihedral angle between two l.s. planes) are estimated using the full covariance matrix. The cell esds are taken into account individually in the estimation of esds in distances, angles and torsion angles; correlations between esds in cell parameters are only used when they are defined by crystal symmetry. An approximate (isotropic) treatment of cell esds is used for estimating esds involving l.s. planes.
Refinement. Refined as a 2-component inversion twin. H atoms were freely refined with *U*_iso_(H) 1.2× or 1.5×*U*_eq_ of the parent atom. The crystal structure was refined as a two-component inversion twin [Flack parameter = 0.374 (5); Parsons *et al.*, 2013].

### (2-Aminoethan-1-aminium-κN2)trichloridozinc(II) Fractional atomic coordinates and isotropic or equivalent isotropic displacement parameters (Å^2^)

**Table:**

	*x*	*y*	*z*	*U*_iso_*/*U*_eq_	
Zn1	0.45714 (2)	0.91365 (2)	0.38549 (2)	0.01331 (3)	
Cl1	0.29829 (4)	0.98873 (3)	0.25628 (2)	0.01659 (5)	
Cl2	0.79364 (4)	0.95070 (4)	0.37517 (2)	0.01909 (5)	
Cl3	0.30307 (5)	1.04462 (3)	0.50133 (2)	0.01698 (5)	
N1	0.42319 (16)	0.66776 (12)	0.40719 (7)	0.01523 (18)	
H1A	0.535 (3)	0.620 (2)	0.3886 (11)	0.018*	
H1B	0.427 (3)	0.642 (2)	0.4615 (12)	0.018*	
N2	0.08625 (17)	0.31486 (12)	0.33981 (8)	0.0185 (2)	
H2A	−0.023 (3)	0.342 (2)	0.3659 (12)	0.028*	
H2B	0.105 (3)	0.209 (2)	0.3455 (12)	0.028*	
H2C	0.074 (3)	0.338 (2)	0.2865 (14)	0.028*	
C1	0.25042 (17)	0.58592 (14)	0.36385 (7)	0.01499 (19)	
H1C	0.260 (3)	0.612 (2)	0.2975 (11)	0.018*	
H1D	0.129 (3)	0.631 (2)	0.3899 (11)	0.018*	
C2	0.26187 (17)	0.40102 (13)	0.38011 (8)	0.01628 (19)	
H2E	0.374 (3)	0.356 (2)	0.3522 (11)	0.020*	
H2D	0.265 (3)	0.3787 (19)	0.4438 (12)	0.020*	

### (2-Aminoethan-1-aminium-κN2)trichloridozinc(II) Atomic displacement parameters (Å^2^)

**Table:**

	*U*^11^	*U*^22^	*U*^33^	*U*^12^	*U*^13^	*U*^23^
Zn1	0.01369 (6)	0.01376 (5)	0.01250 (5)	−0.00133 (4)	−0.00035 (5)	0.00047 (4)
Cl1	0.01636 (12)	0.02048 (10)	0.01292 (10)	0.00025 (10)	−0.00161 (9)	0.00117 (8)
Cl2	0.01381 (11)	0.02772 (12)	0.01575 (11)	−0.00389 (10)	−0.00020 (9)	0.00146 (9)
Cl3	0.01857 (12)	0.01778 (10)	0.01460 (10)	−0.00088 (9)	0.00209 (9)	−0.00156 (8)
N1	0.0162 (5)	0.0142 (4)	0.0153 (4)	−0.0002 (3)	−0.0010 (3)	0.0007 (3)
N2	0.0190 (5)	0.0143 (4)	0.0222 (5)	−0.0001 (3)	−0.0010 (4)	−0.0024 (4)
C1	0.0161 (5)	0.0123 (4)	0.0165 (4)	0.0001 (4)	−0.0016 (3)	0.0011 (4)
C2	0.0162 (5)	0.0128 (4)	0.0198 (5)	−0.0001 (3)	−0.0024 (4)	0.0012 (4)

### (2-Aminoethan-1-aminium-κN2)trichloridozinc(II) Geometric parameters (Å, º)

**Table:**

Zn1—N1	2.0345 (10)	N2—H2A	0.85 (2)
Zn1—Cl3	2.2600 (3)	N2—H2B	0.87 (2)
Zn1—Cl1	2.2646 (3)	N2—H2C	0.81 (2)
Zn1—Cl2	2.2686 (3)	C1—C2	1.5222 (15)
N1—C1	1.4755 (15)	C1—H1C	1.003 (16)
N1—H1A	0.883 (18)	C1—H1D	0.967 (18)
N1—H1B	0.828 (17)	C2—H2E	0.930 (18)
N2—C2	1.4879 (16)	C2—H2D	0.958 (17)
			
N1—Zn1—Cl3	106.99 (3)	C2—N2—H2C	110.9 (14)
N1—Zn1—Cl1	110.15 (3)	H2A—N2—H2C	107.2 (19)
Cl3—Zn1—Cl1	107.313 (12)	H2B—N2—H2C	109.4 (18)
N1—Zn1—Cl2	104.52 (3)	N1—C1—C2	109.68 (9)
Cl3—Zn1—Cl2	115.969 (12)	N1—C1—H1C	106.1 (10)
Cl1—Zn1—Cl2	111.728 (11)	C2—C1—H1C	110.9 (9)
C1—N1—Zn1	117.43 (7)	N1—C1—H1D	108.1 (10)
C1—N1—H1A	109.2 (11)	C2—C1—H1D	110.6 (10)
Zn1—N1—H1A	106.5 (11)	H1C—C1—H1D	111.2 (14)
C1—N1—H1B	109.4 (13)	N2—C2—C1	111.18 (9)
Zn1—N1—H1B	113.2 (11)	N2—C2—H2E	105.8 (11)
H1A—N1—H1B	99.6 (16)	C1—C2—H2E	111.0 (11)
C2—N2—H2A	112.0 (13)	N2—C2—H2D	108.7 (11)
C2—N2—H2B	108.0 (14)	C1—C2—H2D	110.0 (10)
H2A—N2—H2B	109.3 (19)	H2E—C2—H2D	110.1 (16)
			
Zn1—N1—C1—C2	174.46 (7)	N1—C1—C2—N2	178.18 (10)

### (2-Aminoethan-1-aminium-κN2)trichloridozinc(II) Hydrogen-bond geometry (Å, º)

**Table:**

*D*—H···*A*	*D*—H	H···*A*	*D*···*A*	*D*—H···*A*
N1—H1*A*···Cl1^i^	0.883 (18)	2.636 (17)	3.3733 (11)	141.7 (14)
N1—H1*B*···Cl2^ii^	0.828 (17)	2.678 (18)	3.4620 (11)	158.6 (17)
N2—H2*A*···Cl3^ii^	0.85 (2)	2.46 (2)	3.2188 (12)	149.2 (17)
N2—H2*B*···Cl1^iii^	0.87 (2)	2.566 (19)	3.2449 (11)	135.2 (16)
N2—H2*C*···Cl2^i^	0.81 (2)	2.70 (2)	3.4526 (12)	154.2 (19)

Symmetry codes: (i) −*x*+1, *y*−1/2, −*z*+1/2; (ii) *x*−1/2, −*y*+3/2, −*z*+1; (iii) *x*, *y*−1, *z*.
